# Three-dimensional oropharyngeal airway changes after facemask therapy using low-dose computed tomography: a clinical trial with a retrospectively collected control group

**DOI:** 10.1186/s40510-021-00391-3

**Published:** 2021-12-23

**Authors:** Amr H. Husson, Ahmad S. Burhan, Mohammad Y. Hajeer, Fehmieh R. Nawaya

**Affiliations:** 1 Department of Orthodontics, University of Damascus Dental School, Damascus, Syria; 2 Department of Pediatric Dentistry, Faculty of Dentistry, Syrian Private University, Damascus Countryside, Syria

**Keywords:** Maxillary deficiency, Facemask, Oropharyngeal airway, Low-dose computed tomography

## Abstract

**Aims:**

This study aimed to evaluate the short-term oropharyngeal airway volumetric changes in growing Class III maxillary-deficient patients treated by facemask without expansion compared with untreated Class III controls, using low-dose computed tomography.

**Methods:**

Eighteen maxillary-deficient children (9 boys, nine girls) with a mean age of 7.81 ± 0.84 years were treated with maxillary bonded bite block and facemask (FM). Pre- (T1) and post-treatment (T2) low-dose CT images were acquired. Sixteen untreated Class III patients with a mean age of 7.03 ± 0.56 years had previously two low-dose CT scans within a one year of follow-up. Volumetric and minimal cross-sectional area measurements were obtained to assess the oropharyngeal airway changes. Quantitative mean, minimum, and maximum displacement of superimposed 3D models were estimated from a point-based analysis. Paired-samples t-tests were used for the intragroup comparisons, and an independent samples t-test and the Mann–Whitney U tests were carried out for the intergroup comparisons.

**Results:**

A statistically significant increase in the total and retropalatal volumes oropharyngeal airway volume were observed in the control group (302.23 ± 345.58 and 145.73 ± 189.22 mm^3^, respectively). In the FM group, statistically significant increases in the total and retropalatal volumes were observed (738.86 ± 1109.37 mm^3^ and 388.63 ± 491.44 mm^3^, respectively). However, no statistically significant differences were found between the two groups, except for the maximum part analysis which was significantly greater in the FM group (*p* = 0.007).

**Conclusions:**

FM therapy appeared to have no additional effects on the oropharyngeal airway other than those induced by growth.

## Introduction

Although the effects of the protraction facemask (FM) on upper airway have evaluated previously by many studies, the results remain controversial and unclear [1–5]. This can be attributed partially to the use of traditional lateral cephalograms [6, 7], and to the probable effects of the rapid maxillary expansion (RME) in increasing the oropharyngeal airway dimensions when used in conjunction to the FM [8–11]. Isolated effects of the RME in increasing the nasopharynx and oropharynx volumes has been well established [12]. In contrast, the results of studies evaluating the FM-only treatment have been conflicting [1–3, 13]. While Baccetti et al. [1] demonstrated no significant changes in the sagittal upper airway dimensions, Hiyama et al. [3] and Kaygısız et al. [4] showed an increase in the superior upper airway space. Lee et al. conducted a meta-analysis [5] and stressed the need for more 3D cohort studies research with untreated class III controls to determine the potential effects of FM on the upper airway.

The new imaging techniques, such as low-dose computed tomography (CT), is a performed tool in evaluating the upper airway, especially in the transverse dimension [6, 14]. Moreover, volume, surfaces, and cross-sectional area extracted from 3D radiographic imaging by using commercial software offer the possibility to make a more precise evaluation of upper way [6, 7]. Over these measures, the superimposition of 3D model generated from 3D images and point-based analysis explain the changes in size and shape of structures involved in the treatment [15].

To the best of our knowledge, there is no study that has evaluated the volumetric changes of the oropharyngeal airway space following FM-only treatment. Accordingly, the purpose of this clinical study was to evaluate and compare the changes of oropharyngeal airway dimensions after FM-only therapy with those changes induced by growth in a control group of untreated patients using conventional 3D measurements and 3D-model superimposition analysis.

## Methods

### Study design

This study was a non-randomized controlled clinical trial (CCT) study design. The study protocol was reviewed and approved by the Regional Ethical Committee on Research of the Damascus University (UDDS-1091-1900PG). Informed consent was obtained from each patient’s family prior to the patriciate. Funding was provided by the University of Damascus Postgraduate Research Budget (Ref no: 7301144710DEN).

### Sample size calculation

The current study was based on assuming a 1000 mm^3^ difference to be clinically significant between the two groups, and taking into account the observed standard deviation of lower-pharyngeal airway volume 1104.04 in a previous publication [16]. We estimated the need to recruit a sample of 32 children (in the two groups) employing Minitab® v.18.1 software (Minitab, Inc., State College, PA, USA), with a power of 80% and a significance level of 0.05. We added 10% to this value in the experimental group to address the risk of sample attrition. Therefore, the number was raised to 18 children.

### Patients' recruitment and follow-up

Initially, growing patients with ages between 7 and 9 years were screened from those seeking orthodontic treatment at the Department of Orthodontics and Dentofacial Orthopedics, Faculty of Dental Medicine, Damascus University. The clinical inclusion criteria were as follows: Anterior cross-bite or edge-to-edge incisor relationship, Class III relationships of the permanent first molars, normal or deep overbite, straight or concave profile, no temporomandibular joint disorders, and absence of severe maxillary transverse constriction. Twenty patients who met the clinical inclusion criteria were selected and their parents/guardians were approached. The information sheet was given to them and the need for two low-dose CT images was elaborately explained before taking their informed consents. Consequently, maxillary retrognathism was confirmed by lateral cephalograms (N perpendicular to A point <  − 1 mm, 0 ≤ ANB ≥ 4-), and that the growth pattern was normal or horizontal (Bjork´s sum ≤ 401°). Two patients were excluded, one because of a vertical growth pattern and the other because he had only mandibular prognathism. Thus, the final group consisted of 18 patients, 9 boys and 9 girls.

The control group was collected retrospectively, and consisted of 16 untreated class III patients (7 boys and 9 girls) whose low-dose CT records were collected form the database of CT images at the Departments of Orthodontics and Pedodontics. For each patient, the two low-dose CT images were taken with an average of 12 months apart. The rationale for taking their CT images was related to different reasons beyond being classified as Class III skeletal patients, e.g. the presence of supernumerary tooth or misplaced tooth, assessment of the status of the alveolar support, evaluation of the airway competency, or assessment of the nasal cavity structures. The second CT image was captured to assess the progress of the case after approximately one year of the first image. The sample was matched closely for inclusion criteria of the experimental group regarding basically to age, sex, and radiological inclusion criteria.

**Fig. 1:**
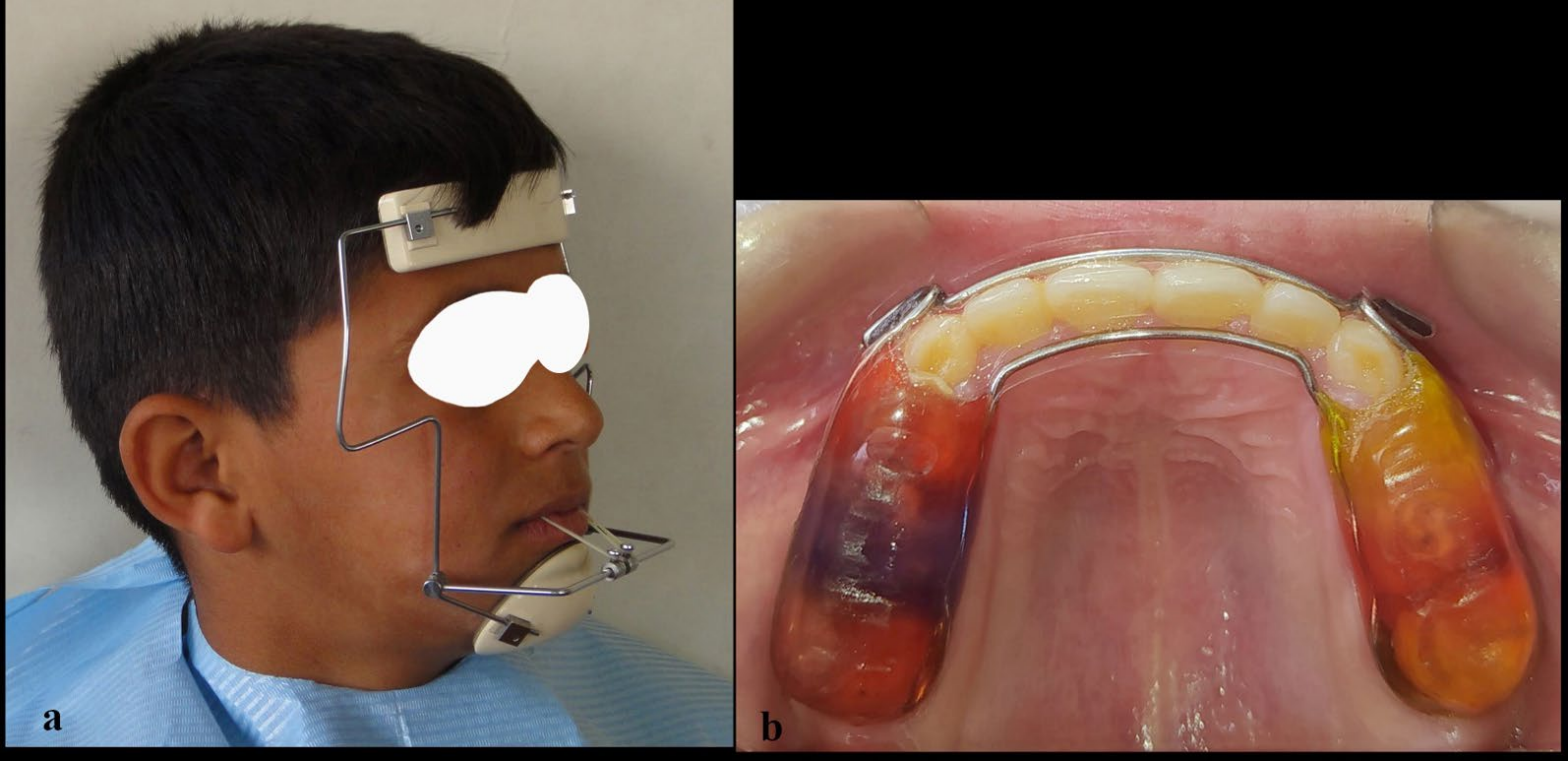
**a** Delaire type, **b** bonded maxillary bite block

**Fig. 2:**
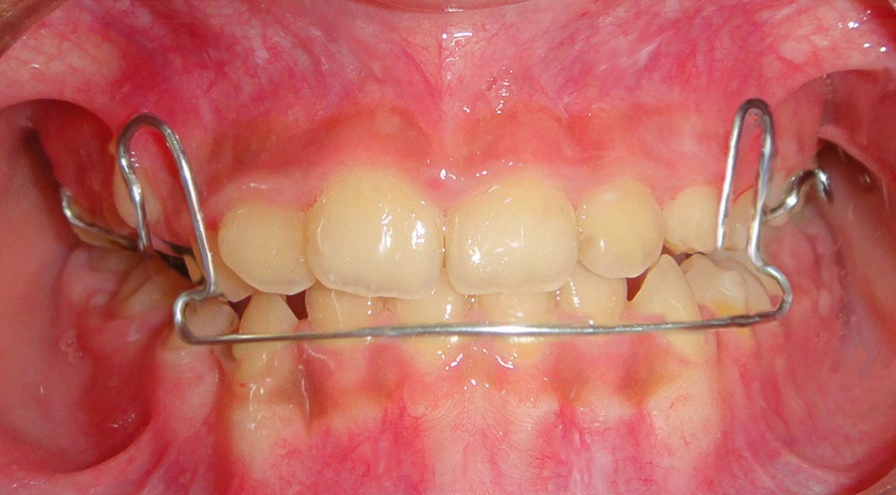
The removable mandibular retractor (RMR) appliance used in this study

All experimental group participants were treated with a modified maxillary bite block and Delaire-type (M0774-00, Leone, Firenze, Italy) facemask (Fig. 1a). The individually fabricated splint consisted of a metal framework of double-soldered buccal and palatal wires to pediatric bands, with vestibular hooks distally to laterals for elastic traction and covered posteriorly with a 2 mm acrylic cap (Fig. 1b). A total of 400 g/side force was applied in a direction approximately 30° inferiorly to the occlusal plane, at least 16 h per day [17]. The active treatment with facemask (FM) therapy was considered 'complete' when three conditions were met: (1) correcting the overjet to achieve three to 4 mm of positive horizontal overlap between incisal edges, (2) achieving a Class I or slightly Class II molar relationship, (3) undergoing the active treatment for not less than 8 months. The removable mandibular retractor (RMR) appliance was used in the second phase to preserve the achieved results. The RMR was a removable appliance that rested on the upper jaw and had an inferiorly extended labial bow that touched the cervical regions of the lower anterior teeth. It has been shown to be an effective appliance in the early correction of skeletal class III malocclusions in patients aged between 5 – 8 year [18] and 9–12 year [19] (Fig. 2).

### Computed tomography acquisition

Two sets of low-dose CT images were acquired, one prior to the treatment (T1) and one at the end of active treatment (T2). The CT scans were taken by one certified radiologist using a Philips Brilliance 64 detectors scanner (Philips Medical Systems, Best, The Netherlands), with the patients were seated in a supine position and their Frankfort horizontal (FH) plane perpendicular to the floor. They were instructed to keep their teeth in maximum intercuspation and their tongue behind the upper incisors during the exposure. The CT parameter values used were suggested by Ballanti [20] as follows; 80 kV, 100 mAs, 1 pitch, 2.5 mGy (CTDIvol), and 1.25 mm slice thickness. All data were stored in the Digital Imaging and Communications in Medicine (DICOM) format and then transferred into MIMICS 21.0 software (Materialise, Leuven, Belgium) for preliminary 3D geometry creation.

**Fig. 3:**
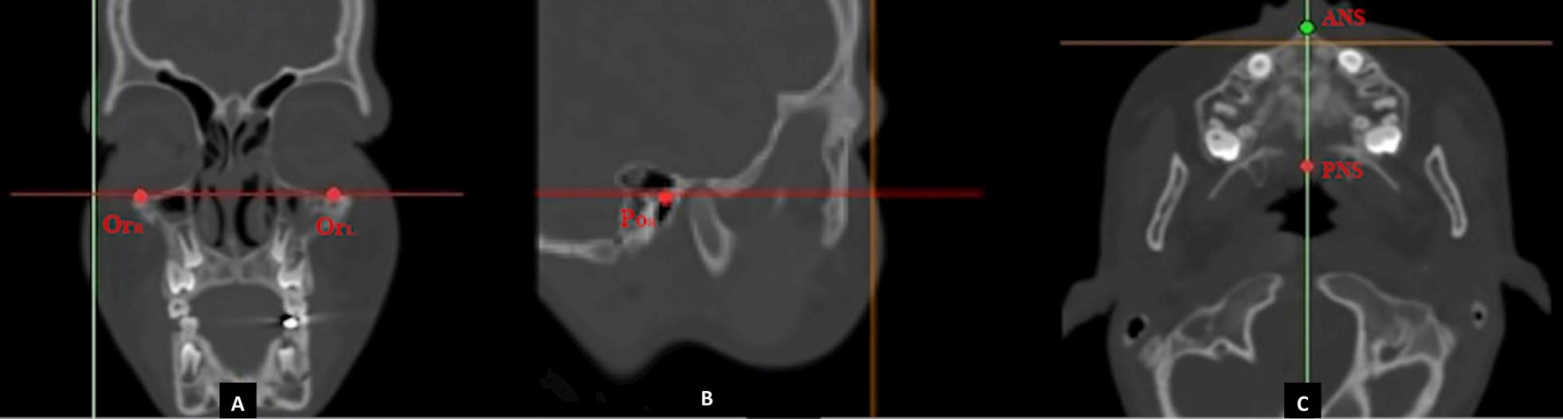
CT image slices of the orientation landmarks: Coronal CT image showing the right orbital Or_R_ and left orbital Or_L_ (**a**). Sagittal view showing right Porion Po_R_ (**b**). Axial view showing anterior nasal spine ANS and posterior nasal spin PNS (**c**)

Each image was exactly oriented as follows: A new reslice plane (RP) was built so that the Frankfort horizontal (FH) plane (defined by right Porion and inter-orbital line in the sagittal and frontal views, respectively) was parallel to the floor, and the axially midsagittal line (defined as the anterior nasal spine to the posterior nasal spine) was perpendicular to the floor (Fig. 3). The image was resliced in the anterior–posterior direction parallel to the FH plane with a 150 mm × 150 mm field of view using the software’s reslice function.

### Segmentation of oropharyngeal airway

**Fig. 4:**
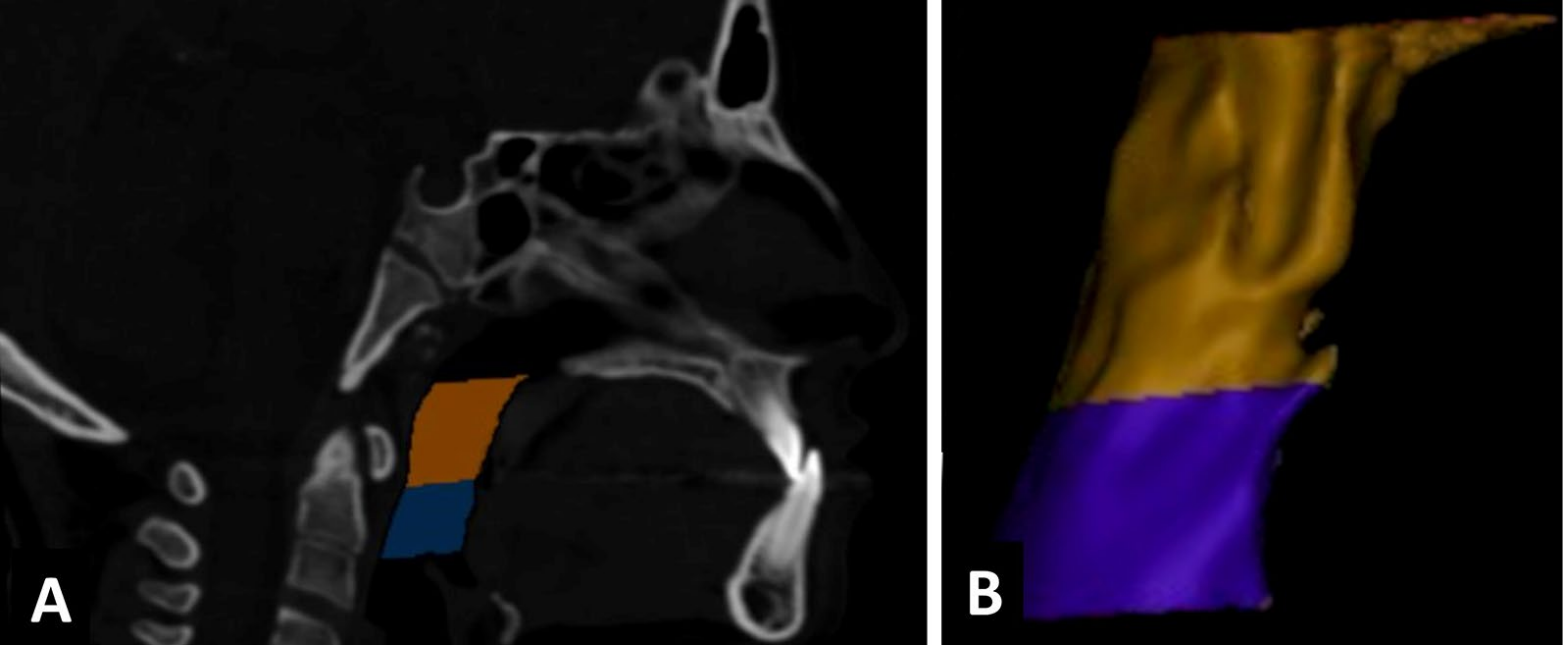
CT midsagittal image slice of the airway segmentation (**a**). Lateral view showing retropalatal and retroglossal 3D parts (**b**)

Semiautomatic segmentation was applied. The upper airway mask was built by threshold tool of the software. Region of Interest (ROI) was selected to cover the upper airway anatomy. The threshold value was adjusted individually between − 1024 and 200 Hounsfield units [HU] to improve the accuracy of the segmentation. Generally, no manual segmentation was implemented. The only manual editing was applied for evident artefacts or uninterested structures like oral cavity. Once an airway mask and a high-quality 3D model was created, the upper airway was segmented in the midsagittal plane in the sagittal view of the software by the orthogonal-to-screen tool (Fig. 4). As in the Chang et al. [21] study, the superior boundary of the upper airway was determined by a plane passing posterior to the nasal spine (PNS) to the Basion (Ba) [P plane], and inferiorly by a parallel plane to EP passing through the most superior point of the epiglottis [EP plane]. Then the upper airway was divided into a retropalatal (upper segment) and a retroglossal airway (lower one) using a parallel plane to the P plane passing the posteroinferior point of the soft palate [SP plane]. (Fig. 4). Finally, the 3D models were smoothed and warped using a voxel-based technique with an existing MIMICS algorithm. The software automatically calculated the total, retropalatal and retroglossal airway volumes. A minimal cross-sectional area was obtained manually and computed using the software’s Area measuring tool.

### 3D superimposition and comparison analysis

**Fig. 5:**
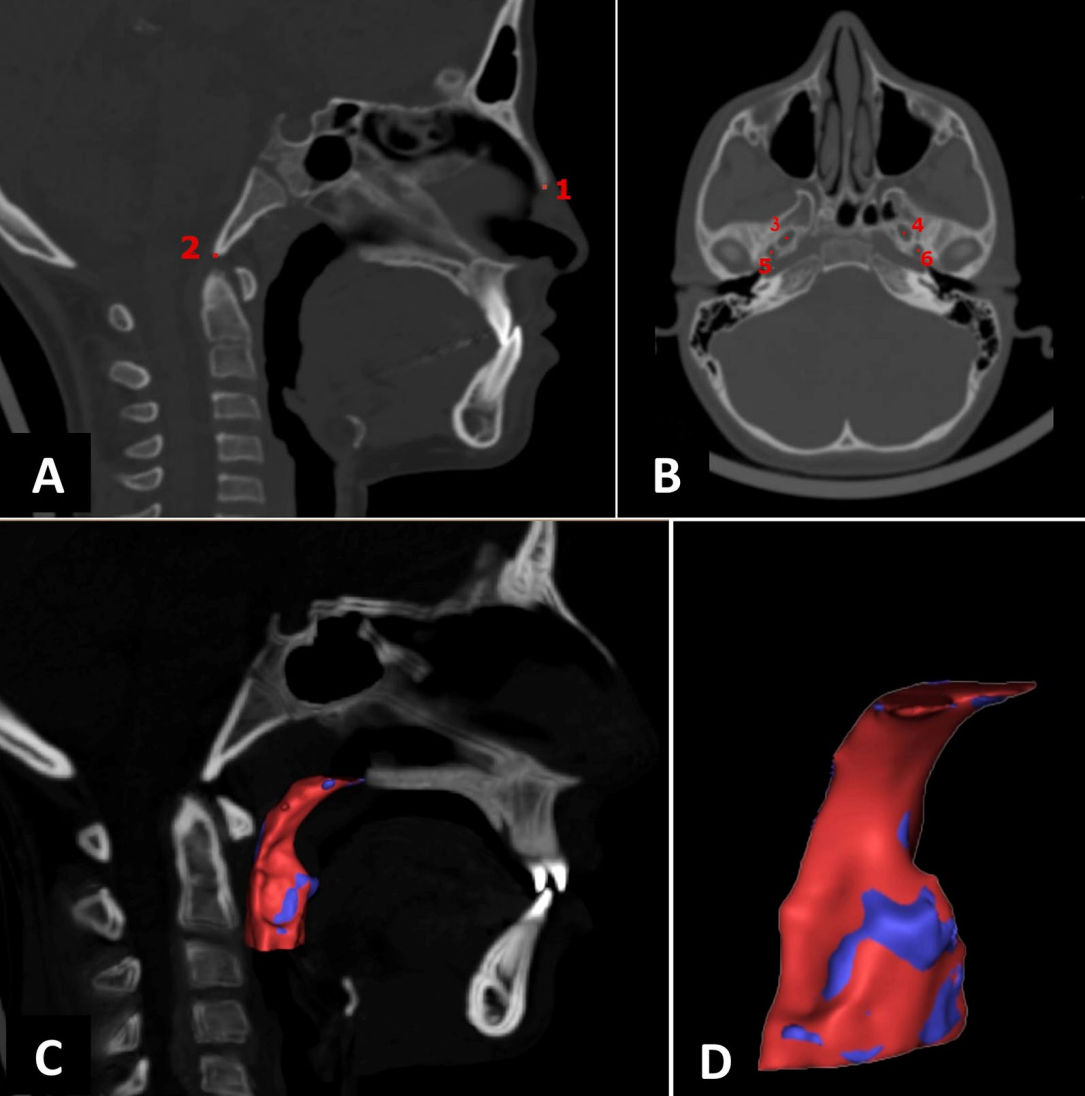
Anatomical landmarks used in low-dose CTs superimposition (image registration, **a** and **b**) and these are: 1. Tip of clivus. 2. Tip of the nasal bone. 3. Right foramen ovale. 4. Left foramen ovale. 5. Right foramen spinosum 6. Left foramen spinosum. Midsagittal slice of registered images and models (**c**). Isometric view of registered T1 (blue airway) and T2 (red airway; **d**)

Landmark-based registration (LBR) was used in this study. The serial resliced projects of each patient containing the CT data set and the 3D models of the upper airway were registered using the image registration tool in Mimics. The six landmarks used in the registration are given in Table 1 and shown Fig. 5. These landmarks were validated in a previous study [22]." The registered 3D models were then exported to 3-matic software (3-matic13.0, Materialise NV, Leuven, Belgium).

**Table 1:** Definition of the anatomical landmarks used in the registration of the pre- and post-treatment images^†^

landmark	Abbreviation	Axial	Frontal	Sagittal
Tip of the nasal bone	NSTP	Mid-inferior radiopaque point of the Inter-nasal suture	Mid-inferior point of the Inter-nasal suture	Anteroinferior point on the nasal bone in the mid sagittal plane
Tip of Clivus	CLVS	Mid-posterior point of the clivus	Mid-inferior point of the clivus	posterinferior point of the clivus in the mid sagittal plane
Right foramen ovale	ROVAL	Mid-point of the inferior complete circle representing the foramen	Mid-point of radiolucent space representing the foramen	Mid-point of radiolucent space representing the forman
Left foramen ovale	LOVAL	Mid-point of the inferior complete circle representing the foramen	Mid-point of radiolucent space representing the foramen	Mid-point of radiolucent space representing the forman
Right foramen spinosum	RSPNM	Mid- point of the inferior complete circle representing the foramen	Inferior mid-point of the opening to infratemporal fossa	
Left foramen spinosum	LSPNM	Mid-point of the inferior complete circle representing the foramen	Inferior mid-point of the opening to infratemporal fossa	

^†^According to Alsufyani et al.[15]

**Fig. 6:**
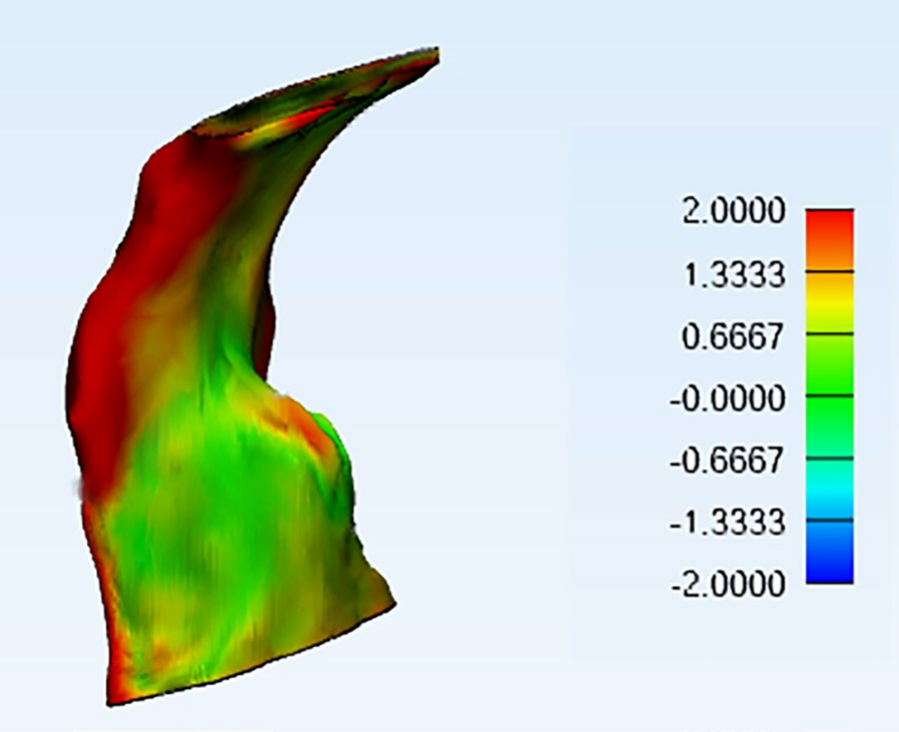
Isometric view of the color-coded map after part analysis between T2 and T1

The 3D comparison between time points T2–T1 was performed using the part comparison analysis tool in 3-Matic software. Details of used tool is described in the work of Alsufyani et al. [22] A color-coded map for each comparison was produced with the threshold set at 2 mm: green areas indicated differences within 2 mm (between − 2 and 2 mm), red surfaces indicated outward (positive values) displacement more than 2 mm between two 3D models), and blue surfaces indicated inward (negative values) displacement greater than − 2 mm (Fig. 6). Mean, minimum, and maximum values of part analyses were reported.

### Statistical analysis

Statistical analyses were carried out using IBM SPSS Statistics for Windows version 26.0 (IBM Corp., NY, USA). The Shapiro–Wilks test showed normal distribution for all parameters except age parameter of the control group. Accordingly, the paired-samples t-test was used for the in-group comparisons, and an independent-sample t-test was used for the intergroup comparisons of parameters with a normal distribution. The Mann–Whitney U was used for inter-group comparison of age. Significance level was set at the 5%.

### Error of the method

All measurements of 17 (25%) randomly selected patients were repeated after one week by the same examiner (AH), and an intraclass correlation coefficient (ICC) (two-way mixed with absolute agreement) was used to assess intra-rater reliability. Errors of the measurements were analyzed with Dahlberg's formula [23].

## Results

The ICCs showed a high level of agreement, ranging from 0.93 to 0.99. The error of the method was 6.09 mm^2^ for area measurement, ranged from 0.11 to 0.23 mm for linear measurements, and from 52.24 to 54.7 for the volumetric measurements (Table 2).

**Table 2:** Reliability of the performed measurements and error of the method

Variable	ICC	95% Confidence Interval		Mean difference*	Errors of the method**
		L.B	U.B		
Total Oropharyngeal airway volume (mm^3^)	0.999	0.999	1.000	9.39	54.7 mm^3^
Retropalatal airway volume (mm^3^)	0.977	0.937	0.991	−10.30	53.35 mm^3^
Retroglossal airway volume (mm^3^)	0.991	0.975	0.997	10.20	52.24 mm^3^
Minimal cross sectional area (mm^2^)	0.989	0.970	0.996	−2.05	6.09 mm^2^
Mean part analysis (mm)	0.986	0.959	0.995	0.02	0.11 mm
Minimum part analysis (mm)	0.991	0.974	0.997	0.01	0.23 mm
Maximum part analysis (mm)	0.992	0.978	0.997	0.06	0.20 mm

*ICC* intraclass correlation coefficients, *L.B* lower bound, *U.B* upper bound

^*^Mean differences between the two assessment times

**Using the Dahlberg's formula. (Dahlberg, 1940)

The two groups’ characteristics, treatment/observation period are given in Table 3. The control group consisted of 7 boys and 9 girls. The mean age was 7.03 ± 0.56 years, and the average observation time was 12.25 ± 1 months. The facemask group comprised 9 boys and 9 girls with a median age of 8 ± 0.84 years. The active treatment period was 11.17 ± 2.18 months. No statistically significant differences between the two groups were found at the beginning of treatment (T1) as shown in Table 4.

**Table 3:** Baseline sample characteristics

Variable	Control group (*N* = 16)		Facemask group (*N* = 18)		Mean difference	*P*-value	95% Confidence interval of the difference	
	Mean	SD	Mean	SD			lower	Upper
Gender(female)^†^	9 (56.25%)	–	9 (50. %)	–	–	0.716	–	–
Age (year)^‡^	7.03	0.56	7.81	0.84	−0.77	0.006**	−1.27	−0.28
Observation/treatment period (month)^§^	12.25	1.00	11.17	2.18	1.08	0.069	−0.09	2.26

*SD* standard deviation

^†^Pearson's Chi-square test

^‡^Mann–Whitney *U* test

^§^Two-sample *t*-test

^**^p < 0.01

**Table 4:** Descriptive statistics and the comparing between the two groups regarding to the baseline measurements^†^

Variable	Control group (*N* = 16)		Facemask group (*N* = 18)		Mean difference	*p*-value^†^	95% Confidence interval of the difference	
	Mean	SD	Mean	SD			Lower	Upper
SNA°	78.02	2.04	78.31	1.70	−0.30	0.654	−1.02	1.6
SNB°	79.22	2.02	79.95	1.42	−0.73	0.231	−0.49	1.94
ANB°	−1.21	0.81	−1.64	0.89	0.44	0.146	−1.03	0.16
SN-POG°	80.97	2.02	81.69	1.42	0.72	0.231	−0.48	1.94
SN-GOME°	33.51	4.16	31.28	3.84	2.23	0.115	−5.02	0.57
Jarabak Ratio	66.95	4.32	65.26	3.22	1.69	0.959	−4.32	0.21
Total oropharyngeal airway volume (mm^3^)	7563.34	2012.41	8308.13	2533.60	−744.79	0.345	−2357.31	867.74
Retropalatal airway volume (mm^3^)	4308.51	1273.48	4767.68	1349.69	−459.17	0.317	−1287.19	604.96
Retroglossal airway volume (mm^3^)	3254.83	930.24	3540.45	1360.66	−285.62	0.408	−1110.52	539.28
minimal cross-sectional area (mm^2^)	163.08	43.53	149.57	27.95	13.51	0.486	−11.75	38.78

^†^Student’s *t* -test

The results of intra-group comparisons indicated a significant increase in the total oropharyngeal airway volumes by a mean of 302.23 ± 345.58 mm^3^ (*p* = 0.003) and 738.86 ± 1109.37 mm^3^ (*p* = 0.012) in the control and facemask groups, respectively. The retropalatal region of the control and the facemask group increased significantly, *p* = 0.008 and *p* = 0.004, respectively. However, no statistically significant differences were found between the T2–T1 changes for the remaining parameters of both groups (*p* > 0.05), as shown in Table 5.

**Table 5:** Descriptive statistics of the CT-based measurements and the *p*-values of significance testing between the two groups

Variable	Control group (*N* = 16)							facemask group (*N* = 18)						
	T1		T2		T2 vs. T1 *p*-value	95% CI		T1		T2		T2 vs. T1 *p*-value	95% CI	
	Mean	SD	Mean	SD		Lower	Upper	Mean	SD	Mean	SD		Lower	Upper
Total Oropharyngeal airway volume (mm^3^)	7563.34	2012.41	7865.57	2110.22	0.003**	118.08	486.37	8308.13	2533.60	9046.98	2740.48	0.012*	187.18	1290.53
Retropalatal airway volume (mm^3^)	4308.51	1273.48	4454.24	1314.12	0.008**	44.90	246.56	4767.68	1349.69	5156.31	1282.47	0.004**	144.25	633.02
Retroglossal airway volume (mm^3^)	3254.83	930.24	3411.33	239.84	0.075	−17.73	330.72	3540.45	1360.66	3890.67	1713.96	0.171	−167.05	867.49
Minimal cross sectional area (mm^2^)	163.08	43.53	164.74	37.71	0.849	−16.56	19.88	149.57	27.95	149.09	34.73	0.925	−11.08	10.12

*95% CI* confidence interval of the difference

*p < 0.05; **p < 0.01

No statistically significant difference was observed between the two groups regarding the volumetric changes in the oropharyngeal airway and minimal cross-sectional area measurements, as shown in Table 6. The average maximum part analysis of the point-based analysis was significantly greater in the FM group than the control group (*p* = 0.007). However, there was no statistically significant difference in the average mean and minimum part analysis between the two groups (Table 6).

**Table 6:** Descriptive statistics of the changes observed between T2 and T1 and the *p*-values of the statistical tests

Variable	Control group (*N* = 16)		Facemask group (*N* = 18)		Mean difference	*p*-value	95% CI	
	Mean	SD	Mean	SD			Lower	Upper
Total Oropharyngeal airway volume (mm^3^)	302.23	345.58	738.86	1109.37	−436.63	0.128	−1009.94	136.68
Retropalatal airway volume (mm^3^)	145.73	189.22	388.63	491.44	−242.90	0.065	−242.90	125.12
Retroglossal airway volume (mm^3^)	156.50	326.96	350.22	1040.18	−193.72	0.462	−193.72	258.44
Minimal cross sectional area (mm^2^)	1.66	34.20	−0.48	21.32	2.14	0.826	−17.53	21.81
Point-based analysis								
Mean part analysis (mm)	0.25	0.30	0.58	0.61	−0.33	0.54	−0.67	0.01
Minimum part analysis (mm)	−2.38	1.13	−2.94	1.29	0.56	0.192	−0.30	1.41
Maximum part analysis (mm)	3.14	1.51	4.80	1.80	−1.67	0.007**	−2.82	−0.51

*95% CI* 95% Confidence interval of the difference

**p < 0.01

## Discussion

Previous studies into the effects of a protraction facemask on the upper airway dimensions have poor evidence and conflicting outcomes due to using 2D imaging to evaluate this complex anatomical region and having no control samples [3, 5, 9, 13, 20]. Addressing this limitation requires the use of a 3D imaging technique with the presence of an untreated control group.

Obtaining an ethical approval to recruit patients in the control group where no treatment would be provided was deemed difficult. Therefore, Class III patients in the control group were selected from the archives of the Departments of Orthodontics and Pediatric Dentistry who had been referred to the Radiographic Department for CT imaging. On the other hand, patients in the experimental group were intentionally imaged using the same low-dose imaging apparatus. In this context, the low-dose CT protocol has been used in previous published papers and it has been advocated as an alternative method from the conventional CT scanning with a mean absorbed dose similar to that related to conventional radiographic exams for an ordinary orthodontic patient [14, 20]. Therefore, in the current study, the CT radiation exposure was deemed acceptable and below the threshold for harm [24]. In this study, the FM was used only in non-maxillary transverse constriction patients, understanding that RME showed no improvement in maxillary protraction results [25]. Moreover, RME has been established to increase the size of the upper airway [12], and this may affect the accuracy of the results.

The end point of our evaluation was at the end of the active treatment (i.e., T2). Of course, it would be more beneficial to the treating orthodontist if there were some measures of the post-traction changes in the short and long run when using this appliance following FM therapy. However, this was not the objective of the current study. The main changes of the RMR when used in the early treatment of skeletal Class III patients include: 1) an anterior morphogenetic rotation of the mandible, (2 an increase in the maxillary length, and (3) a decrease in mandibular dentoalveolar protrusion [18]. Therefore, the use of the RMR following the active phase may have affected the oropharyngeal airway space in a way or another, but this issue requires additional research work.

Definition of upper airway plays a foundation role in the volumetric assessment. Many methods were suggested previously [6, 11, 16, 21], but the points and planes used in this study were least affected by the patients’ supine position and the neck posture. Moreover, the nasopharynx was not included in this study, because of the potential difference in volumetric assessment due to particular reaction and growth pattern of the nasopharynx's adenoid tissue [13, 26].

There are three main methods for serial image registration: landmark-based, surface-based, and voxel-based techniques. Every technique has inherent limitations, advantages, and disadvantages. Furthermore, all these techniques have been suggested in the literature to work properly [27]. The superimposition method used in this study has been considered as a validated method based on a previous study [22]. The landmarks used in the superimposition have been considered anatomically stable structures by the age of five years as 85% of growth is completed in this area [28]. This method has been used in previous studies interested in the upper airway regions [15, 22]. Moreover, the reliability test of the measurements extracted from the fusion 3D models (i.e., mean part analysis (mm), minimum part analysis (mm), and maximum part analysis (mm)) showed a high level of agreement (ICCs were greater than 0.959) in the current investigation. Additionally, the error of the method regarding these parameters was very small and less than 0.25 mm (ranged between 0.11 and 0.23 mm). Many other benefits could be seen beyond the use of the superimposition in this study, such as the location and distribution of the changes of the upper airway induced by the growth, and the facemask [15]. Therefore, superimposition and part comparison analysis (point-based superimposition analysis) have been used in this study in addition to the volumetric and cross-sectional area measurements.

Although there was a significant difference between the two groups regarding the mean age (*p* = 0.006), the other baseline characteristics of the included patients, such as the sex distribution, and the observation period were found generally homogenous (Table 3). It is worth mentioning that the comparisons made in the current study were focused on evaluating changes (T2–T1) in the first group against changes (T2–T1) in the second group. In other words, no comparison was made between T2 values in the second group versus T2 values in the first group. By comparing change versus change, any discrepancy that could have existed between the two groups at T1 would not affect the validity of the comparisons made. Moreover, the comparison between the two groups at the baseline data regrading to the severity of the sagittal discrepancy, growth pattern, volumetric and superficial values showed statistically insignificant differences between the two groups (Table 4). This meant that the intervention and control arms were comparable in the current study.

The average active treatment time in the treatment group was months 11.17 (standard deviation of 2.18). Some patients had a minimum of 9 months of treatment, whereas 12 patients had a full one-year of active treatment. On the other hand, the average observation time in the control group was 12.25 months. The difference between the two groups regarding the observation period was statistically insignificant (*P* = 0.069). Generally, the mean observation period in the control group can be considered relatively enough to detect possible small changes that should be attributed to growth. However, growth effects can be better demonstrated if longer periods of observation was implemented in the current study.

The mean increase in the airway volume produced by growth was 302.23 mm^3^, which was less than the amount of expected growth of 897.2 mm^3^ mentioned in a previous study [29]. Pamporakis et al. [11] attributed the difference to inhibition development of airway in class III patients compared to normal ones. Another reason may relate to the limitation of cross-sectional study results, which were considered an airway volume-related age study.

After FM treatment, total upper airway and retropalatal volumes increased significantly (i.e., a mean of 738.86 mm^3^ and 388.63 mm^3^, respectively). However, the volume of the retroglossal region increased insignificantly. Unfortunately, the previous studies on the non-expansion FM therapy assessed the airways two-dimensionally using cephalograms [1, 4, 9, 13]. The sample without expansion from the Mucedero et al. [9] study and the study of Baccetti et al. [1] were the closest to our study in terms of mean age and mean treatment time. They reported that the FM caused no sagittal changes in the oropharyngeal and nasopharyngeal regions, which contrasts with our results. The results of Baloş et al. [13] and Danaei et al. [2] indicated a positive alteration in the nasopharyngeal space after FM treatment, which were similar to the current findings. Hiyama et al. [3] reported that the superior upper airway dimension was probably influenced by the maxillary protraction, although that study found non-statistically significant results. In fact, the direct comparison between 2 and 3D measurements is difficult [5].

Interestingly, Pamporakis et al. [11] showed no statistically significant difference in the lower total airway volume (the “total volume” in the current study) after RME/FM treatment. The segmentation method of the airway may be a reason for the difference. Another possible reason is the respiration phase effects due to the wide range of capture time from 7.8 to 40 s [21]. In the current study, no statistically significant differences were observed between growth or FM and growth-induced changes regarding the total upper airway and its regions. The current results were in agreement with previously mentioned studies with untreated class III patients [1, 9].

**Fig. 7:**
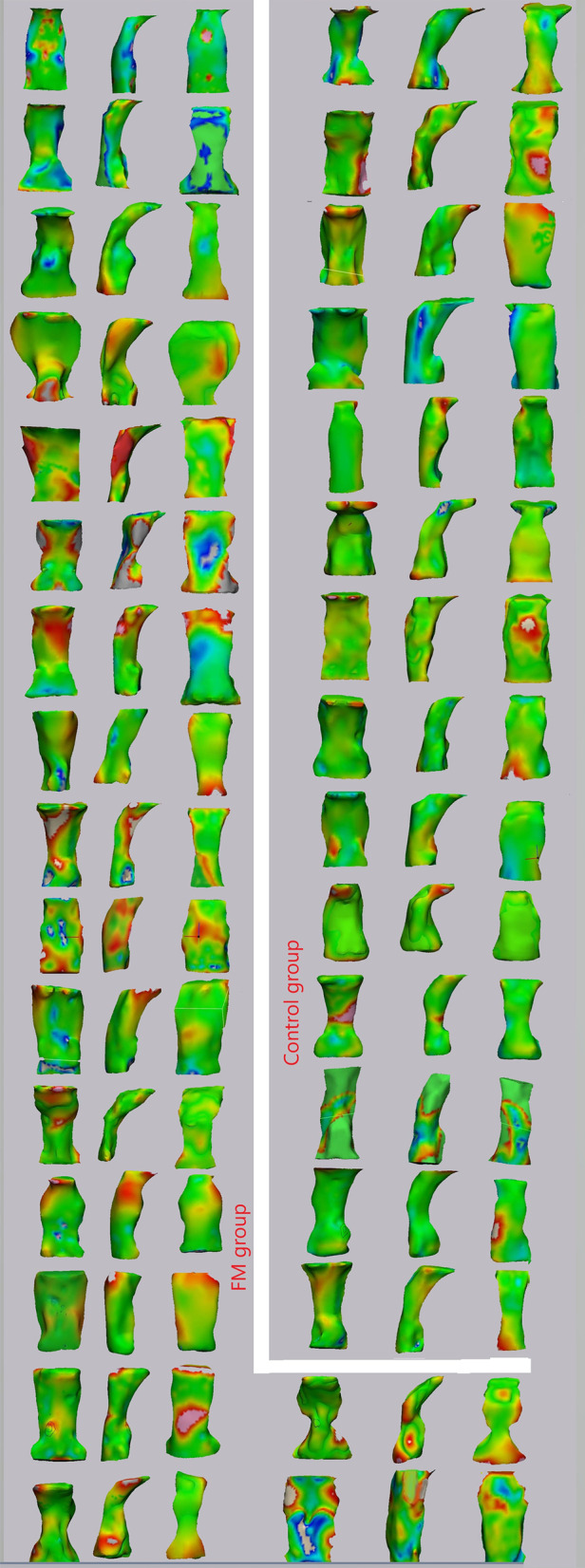
Frontal, lateral and back views of point-based analysis color maps

Qualitatively, some changes were noticed in the upper airway shape indicated by the red and blue areas in the color mapping from the superimposition of pre- and post-treatment models shown in Fig. 7.

Quantitative results of the point-based analysis showed positive mean displacement in both control and FM groups, at 0.25 ± 0.30 mm and 0.57 ± 0.61 mm, respectively. In other words, most triangle nodes of the tested model at T2 were outside of matched triangle nodes from the reference model at T1. According to the results of the point-based analysis, the observed changes due to growth (i.e. the control group) or growth + facemask therapy (i.e. the experimental group) increased the oropharyngeal airway in similar amounts. However, these amounts were clinically insignificant. Intergroup comparisons, according to the maximum point part analysis, showed significant differences between the two groups. The difference might be explained by the effects of neck flexion on this measure. It was evident that the head position was reproducible for a 2-year period [30]. On the other hand, Yagci et al. [31] proved a significant cranial flexion of about 6.4 degrees after 1-year treatment of 45 patients: 9.6 ± 1.4 years of age by FM/RME. Moreover, Alsufyani et al. [22] found a strong positive correlation between the minimum/maximum part analysis results and distance from the second cervical vertebrae to the third. Contrary, volumetric measures showed weak correlations. Accordingly, the potential difference in the registration of T1 and T2 between the two groups might be the reason for the difference.

The limitations of this study were the absence of randomization, long-term observation after the retention phase of the RMR, and respiratory functional examination. However, the volumetric measurements and the quantitative data of the superimposition explained the actual effects of FM on upper airway space.

## Conclusions

- FM therapy appeared to have no other effects on the upper airway than those induced by growth only.

## Data Availability

The datasets used and/or analyzed during the current study are available from the corresponding author on reasonable request.

## Abbreviations

FM: Facemask
RME: Rapid maxillary expansion
CT: Computed tomography
